# Cardiovascular biomarkers in body fluids: progress and prospects in optical sensors

**DOI:** 10.1007/s12551-022-00990-2

**Published:** 2022-08-18

**Authors:** Reena V. John, Tom Devasiya, Nidheesh V.R., Sphurti Adigal, Jijo Lukose, V. B. Kartha, Santhosh Chidangil

**Affiliations:** 1 Centre of Excellence for Biophotonics, Department of Atomic and Molecular Physics, Manipal Academy of Higher Education, Manipal, Karnataka India 576104; 2 Department of Cardiology, Kasturba Medical College, Manipal, Manipal Academy of Higher Education, Manipal, Karnataka India 576104

**Keywords:** Cardiovascular Diseases, Biomarkers, Omics methods, Spectroscopy techniques

## Abstract

Cardiovascular diseases (CVD) are the major causative factors for high mortality and morbidity in developing and developed nations. The biomarker detection plays a crucial role in the early diagnosis of several non-infectious and life-threatening diseases like CVD and many cancers, which in turn will help in more successful therapy, reducing the mortality rate. Biomarkers have diagnostic, prognostic and therapeutic significances. The search for novel biomarkers using proteomics, bio-sensing, micro-fluidics, and spectroscopic techniques with good sensitivity and specificity for CVD is progressing rapidly at present, in addition to the use of gold standard biomarkers like troponin. This review is dealing with the current progress and prospects in biomarker research for the diagnosis of cardiovascular diseases.

Expert opinion.

Fast diagnosis of cardiovascular diseases (CVDs) can help to provide rapid medical intervention, which can affect the patient’s short and long-term health. Identification and detection of proper biomarkers for early diagnosis are crucial for successful therapy and prognosis of CVDs. The present review discusses the analysis of clinical samples such as whole blood, blood serum, and other body fluids using techniques like high-performance liquid chromatography-LASER/LED-induced fluorescence, Raman spectroscopy, mainly, optical methods, combined with nanotechnology and micro-fluidic technologies, to probe patterns of multiple markers (marker signatures) as compared to conventional techniques.

## Introduction

The American Heart Association (AHA), in its latest report, has published up-to-date statistics related to heart disease, stroke, and cardiovascular risk factors which include core health behaviors (smoking, physical activity [PA], diet, and weight) and health factors (cholesterol, blood pressure [BP], and glucose control) (Tsao et al. 2022). Cardiovascular diseases (CVD-s) produce immense health and economic burdens and are the number 1 “Killer” disease all over the world. The term CVD-s includes a range of major clinical heart and circulatory disease conditions (stroke, congenital heart disease, rhythm disorders, subclinical atherosclerosis, coronary heart disease, heart failure [HF], valvular heart disease, venous disease, and peripheral artery disease) and results in a humongous healthcare burden costing hundreds of billions of dollars, not including the cost of nursing home care and billions lost in productivity (indirect costs) due to premature CVD mortality. (Tsao et al. 2022) (Mokou et al. 2017).

The prevention of CVD demands maintaining a proper lifestyle and monitoring of risk factors like inflammation, diabetes, etc. (Satoh et al. 2019). The early diagnosis of CVD has a pivotal role in the patient health. Preliminary diagnosis of acute coronary syndrome (ACS) is carried out depending upon the four factors: (i) clinical history, including age, cardiac history, sex, etc., (ii) physical examination, which includes pale skin color, hypotension, cool skin, etc., (iii) ECG variation, and (iv) cardiac biomarker test. The diagnosis will be confirmed for the possible ACS patients using the methods such as echocardiogram (ECHO), treadmill test (TMT), coronary angiography (CAG), etc. The detection of a suitable biomarker in body fluids has high potential in further evaluation of the severity of the disease and can be very useful for improving the accuracy of CVD diagnosis and in prognosis, therapy planning, and follow-up (A.J. et al. 2013). The biomarkers may also help in understanding the pathophysiology of diseases (Sahu et al. 2011). As per the World Health Organization (WHO), a biomarker is stated as “almost any measurement reflecting an interaction between a biological system and a potential hazard, which may be chemical, physical, or biological. The measured response may be functional, physiological, biochemical at the cellular level, or a molecular interaction” (Strimbu and Tavel 2010). An ideal biomarker is expected to be highly sensitive, specific, and cost effective. A novel biomarker can be validated depending upon its features like sensitivity, accuracy, reproducibility in desired procedure specific to the particular outcome, easy to be used by clinician, and acceptable by the patient (Vasan 2006; Dhingra and Vasan 2017). There are two approaches for biomarker detection; one is knowledge based and the other is unbiased detection. The knowledge-based approach includes an understanding of the biological process and also improving the existing assay methods for the detection of known biomarkers. The unbiased approach includes the application of existing technologies to scroll over the molecules for biomolecular profile characterization of the disease state. These two approaches are complementary to each other (Vasan 2006). The optical methods discussed below can be useful for both approaches.

## Cardiac biomarkers

There are biomarkers, useful for the diagnosis, prognosis, monitoring of the CVD, and treatment follow-up by monitoring the outcome of specific therapies. They can also help the clinician in assessing the risk factors (Dhingra and Vasan 2017; X. Wang et al. 2020a, b). The history of cardiac biomarkers started from aspartate aminotransferase (AST) to the troponins, which are currently recognized as the gold standard biomarker. The adverse outcomes of elevated cardiac biomarkers such as CK-MB and myoglobin in coronavirus disease-19 (COVID-19) patients have also been reported (Yang et al. 2021). Multi-marker detection is, at present, gaining attention due to minimization of error in comparison with single biomarker measurements (Panteghini 2004). Measuring multiple biomarkers is found to be beneficial when compared with the single biomarker-based modalities (A., J.P., and H. 2016).

AST, lactate dehydrogenase (LDH), creatine phospho-kinase (CPK), and myoglobin were recognized as suitable biomarkers for CVD even from the early days. AST was the first biomarker identified for acute myocardial infarction (AMI). The level of AST is elevated in the blood in 3–4 h after the occurrence of AMI, peaks within 28 h, and comes to a base value within 5 days. The elevated level of AST is also found in hepatic congestion, myocarditis, pericarditis, pulmonary embolism, shock, electrical cardioversion, and tachyarrhythmias. AST exists in two isoforms in tissue, one in mitochondria (m-AST) and second in cytoplasm (c-AST). Among the isoforms of AST, M-AST was more suitable for the detection of MI (Yardim 2019; Danese and Montagnana 2016).

Lactate dehydrogenase (LDH) is also proposed as a marker for AMI. Among the five isoforms of LDH, the ratio of LDH1 to LDH2 is reported to be specific for MI. LDH is elevated in the blood within 10 h after the occurrence of AMI; its value peaks within 144 h and comes to baseline value within 12 days. Other than the heart, LDH is expressed in organs like the liver, lung, erythrocytes, and kidney, thus making it less specific (Garg et al. 2017; Danese and Montagnana 2016).

Creatine phospho-kinase is known to be a biomarker for cardiac injury. Its level increases in the blood within 3–9 h after the occurrence of AMI, increases within 20 h, and attains baseline within 72 h. Later, its relation to the severity of MI and prognosis value was discovered. It lacks specificity due to its presence in the liver, kidney, skeletal muscles, and biliary tract diseases. It is available in three isoforms, CK-MB, CK-MM, and CK-BB, originating from muscle (M) and brain (B). According to the studies, it is found that CK-MB is more specific to cardiac injury compared with other isoforms of CK. CK-MB activity is also associated with skeletal muscle disorders thus having less specificity (Lewandrowski, Chen, and Januzzi^ 2002^; Hedges R. Jerris, Hoekstra W. James 1996; Kleiman et al. 2002).

Myoglobin level may increase within an hour of myocardial cell death and is used to know the extent of MI. Its concentration peaks within 4 to 6 h and return to a base value in a day and a half in the blood. Since it clears from the blood rapidly, it is not useful for patients who are presented late. It is also found in skeletal muscular dystrophy, inflammation (myositis) or the presence of acute or chronic renal failure, and trauma thus reducing specificity. It can also increase in the case of strenuous exercise, muscle injections, and in the presence of drugs or toxins (Moe and Wong 2010; Brian Gibler et al. 1987; Lewandrowski, Chen, and Januzzi 2002).

Cardiac troponin is now recognized as the “Gold Standard” for many cardiac conditions (20–23). The importance of troponin for cardiovascular disease diagnosis has been recognized since 1987. Cardiac troponin has 3 isoforms, troponin T which attaches troponin complex to actin filament; troponin C has a calcium-binding site; and troponin I inhibits the interaction of myosin heads in the absence of calcium ions. The cardiac and skeletal muscles are synthesized by troponin C. Troponin I and T are also called cardiac troponins due their high specificity to cardiac myocytes (Jeremias and Gibson 2005). The half-life of troponin in plasma is about 2 h. The peak value of troponin is at 12 h and remains for 10 days or more. The extent of myocardial injury and likelihood of being affected by AMI can be quantitatively known by troponin measurement. Troponin T can help to predict coronary disease even in the presence of renal dysfunction (Aviles, R.J., A SKARI T. A RMAN T. 2002). If the troponin value is negative in highly sensitive troponin assays during patients’ presentation to the emergency department, and persists the same for 3 h, then 99% of patients can be excluded from the AMI prediction. Troponin is released to the circulatory system due to many factors such as apoptosis, normal cell turnover, myocyte necrosis, etc. The sensitivity of troponin depends upon cause of its release to circulatory system and technique with which it is being detected. It cannot differentiate between type I and type II myocardial infarction. Elevation of troponin can be found in patients with pulmonary embolism, sepsis or hypertensive emergencies thus making it less sensitive (Allahham, Singh, and Jneid 2019) (Bucciarelli-Ducci et al. 2004). Table 1 gives an account of currently known biomarkers of CVD.

**Table 1:** Marker proteins, their sources, and the diseases

Name	Source	Target	Details	Reference
Adiponectin	Serum	Coronary sclerosis and ACS	Low circulating adiponectin is found to be associated with coronary sclerosis and ACS. Lower level of adiponectin is also related to metabolic syndrome, type-2 diabetes, and obesity	(Urbonaviciene et al. 2010)
Adipsin	Serum	CAD	–	(Satoh et al. 2019)
Adrenomedullin	Plasma	Heart failure	Release of adrenomedullin is related to the severity of heart failure. It is inversely related to the left ventricular ejection fraction. Detection of adrenomedullin in plasma is difficult due to rapid clearance in the circulation	(Toshio and Yasuaki 2017)
Albumin	Serum	Cardiovascular mortality	Lower concentration of albumin is linked with higher level of cardiovascular mortality	(Anderon 2005)
Angiopoietin-2	Plasma	MI	Plasma level of angiopoietin-2 is increased in patients having high risk of MI. It is also a maker for non-small cell lung cancer and breast cancer	(Patel et al. 2008)
Angiotensin-converting enzyme	Blood	Stroke	Lower level of ACE is an indicator of stroke	(Brscic et al. 2000)
Annexin A2	Circulating biomarker	HF	Is a circulating biomarker for cardiomyopathy-induced heart failure	(Kontush 2016)
Apolipoprotein A-1	Plasma	MI	Decreased level of apolipoprotein A-1 is found in MI patients	(Májek et al. 2011)
Apolipoprotein A-IV	Serum	Risk for CHD	Apolipoprotein A-IV independently predicts risk for CHD	(Tuñón et al. 2016)
Apolipoprotein E	Blood	AMI	Apolipoprotein E is a predictor of adverse events	(Brscic et al. 2000)
Apolipoprotein C-III	Blood	CHD	Apolipoprotein E is a CHD marker independent of cholesterol	(Alaaraji 2019)
Aspartate aminotransferase	Blood	AMI	The elevated level of AST is also found in hepatic congestion, myocarditis, pericarditis, pulmonary embolism, shock, electrical cardioversion, and tachyarrhythmias	(Daniel and Leong 2010a)
B-type natriuretic peptides	Plasma	CAD and MI	BNP level indicates the severity of the ischemia. BNP may be used as a prognostic biomarker for heart failure	(Heeschen et al. 2004)
Cardiac myosin-binding protein C	Circulating	NSTEMI	–	(Kontush 2016)
Cathepsin B	Circulating	Vulnerable plaque	–	(J. Chen et al. 2002)
Cathepsin G	Plasma	Atherosclerotic	The plasma level of cathepsin G is reduced in atherosclerotic patients	(Lynch et al. 2016)
Cathepsin K	Plasma	CAD	Increased plasma level of cathepsin K predicts CAD	(Lynch et al. 2016)
CD 40	Plasma	ACS	CD 40 has a role in the inflammatory process and atherosclerosis. Elevated CD-40 expression is linked with diabetes, which is one of the risk factors for arteriosclerosis	(Heeschen et al. 2003)(Aggarwal et al. 2004)
CD59	Plasma	CAD	–	(Kontush 2016)
CD5L	Plasma	MI	–	(Kontush 2016)
CD 105	Plasma	Stroke and MI	–	(Anderon 2005)
Ceruloplasmin	Plasma	CVD		(Anderon 2005)
Chitotriosidase	Plasma	Atherosclerosis	Increased level of chitotriosidase is found in patients having atherosclerosis	(Anderon 2005)
Choline	Serum	ACS	Choline is associated with coronary plaque vulnerability and ischemia. In troponin-positive patients, serum level choline can differentiate between low-risk and high-risk patients	(Danne and Möckel 2010)
Coagulation factor VII	Serum	CVD	Coagulation factor VII activating protease indicates the increased risk to CVD	(Frishman 2002)
Collagen III	Serum	AMI and CAD	Increased level of collagen III in serum is the predictor of AMI and CAD	(Lynch et al. 2016)
Complement C1	Serum	MI	–	(Muscari et al. 1995)
Creatine kinase	Blood	MI	It lacks specificity due to its presence in the liver, kidney, skeletal muscles, and biliary tract diseases	(Hedges R. Jerris, et al. 1996)(Kleiman et al. 2002) (Brogan et al. 1997)
C-reactive protein	Plasma	Vascular inflammatory, atherosclerosis and CVD, ACS	C-reactive protein has longer plasma half-life. In comparison with healthy samples, the plasma levels of CRP are found to be increased in ACS patients	(Mora et al. 2006)(Ridker 2003)
C-terminal–provasopressin (copeptin)	Blood	AMI	Studies have shown the prediction of heart failure after AMI by copeptin. It is also a marker for neurohormonal stress making it less specific to CVD	(Daniel and Leong 2010b)
Cyclophilin A	Plasma	MI	Plasma levels of cyclophilin A are found to be positively associated with MI	(Kontush 2016)
Cystatin-C	Plasma	CHD	–	(Koenig et al. 2005)
D-dimer	Plasma	Thrombosis and fibrin degradation	D-dimer is a marker for thrombosis and fibrin degradation	(Fareed et al. 1998)(Ottani and Galvani 2001)
Defensin 5	Circulating	CAD	–	(Kontush 2016)
Desmin	circulating	HF	–	(Kontush 2016)
Emilin 3/multimerin-2	Plasma	CAD	–	(Kontush 2016)
Endothelial leucocyte adhesion molecule 1	Plasma	Stroke	–	(Anderon 2005)
Endothelin-1	Circulating	MI	Its levels increased in MI. It can predict heart failure followed by AMI. It is very unstable, thus often binds with other proteins and receptors. Thus, its concentration is difficult to measure	(Daniel and Leong 2010a)
Enolase, beta	Serum	AMI	Its level is elevated significantly in AMI	(Colony 1987)
FAS, soluble	Plasma	Future CVD	Future CVD may be predicted by its elevated levels in plasma	(Troyanov et al. 2003)
Fibrinogen	Serum	MI	It is a risk predictor for CVD. Studies suggest the level of fibrinogen can reflect the prothrombotic and inflammatory state. Increased serum level of it has been found in MI patients	(Muscari et al. 1995)
Fibrinogen gamma chain	Plasma	CAD	–	(Kontush 2016)
Fibrinopeptide A	Plasma	ACS	Higher concentration is found in ACS patients	(Fareed et al. 1998)
Fibrinopeptide B beta 1–42	Plasma	Recurrent ischemia	It may be a predictor of recurrent ischemia	(Anderon 2005)
Fibrinopeptide B beta 15–42	Plasma	Candidate hemostasis	–	(Anderon 2005)
Fibronectin	Circulating	Endothelial cell activation	–	(Lynch et al. 2016)
Galectin-3	Circulating marker	Cardiovascular death	Higher level of galectin-3 is found to be a predictor of cardiovascular death	(Alexander 2018)
Gamma-glutamyltransferase	Plasma	Stroke	It is a marker for stroke but it is also a marker for liver dysfunction and alcohol intake	(Anderon 2005)
Glutathione peroxidase-1	Circulating	Cardiovascular diseases according to the studies in patients with CAD	The red blood cell glutathione peroxidase-1’s decreased activity is an indication of cardiovascular diseases according to the studies in patients with CAD	(Bonaterra et al. 2010)
Glycogen phosphorylase BB	Plasma	ACS	Its level increases in 2–4 h after ischemia, returns to normal value in 1–2 days	(Peetz et al. 2005)
Growth differentiation factor-15	Circulating marker	Recurrent MI, in NSTEMI patients	It is released in response to inflammation and oxidative stress. It is a cytokine responsive to stress. It may be used as a prognostic marker in MI	(Andersson et al. 2016)
Growth hormone	Serum	Cardiovascular death	It is linked with an increased occurrence of cardiovascular death	(Vahl et al. 1999)
GST-omega-1	Plasma	CAD		(Kontush 2016)
Heart-type fatty acid binding protein	Serum	Acute ischemic strokes and heavy exercise	It is a cytoplasmic protein. In case of myocardial injury, cytoplasmic proteins along with H-FABP are released into the circulation. The higher level is observed in acute ischemic strokes and heavy exercise	(Okamoto et al. 2000)
Heat shock protein-27	Plasma	CAD	Plasma level of it is diminished in the case of MI and CAD	(Kontush 2016)
Heat shock protein-60	Blood	CVD		(Rizzo et al. 2011)
Hepatocyte growth factor	Serum	AMI		(Sato et al. 1997)
HDL-C	Plasma	CVD	HDL-C is associated with decreasing vascular inflammation and thrombosis, promoting endothelial repair and improving endothelial function	(Hoefer et al. 2015)
Homocysteine	Serum	Non-ST-elevated ACS	Serum level of homocysteine level is found elevated in non-ST-elevated ACS. Some of the cardiac risk factors such as old age, lack of exercise, high blood pressure, smoking, and high cholesterol is linked with homocysteine	(Bodí et al. 2005)
Hydroxybutyrate dehydrogenase	Plasma	Infarction size in MI	It is a mitochondrial enzyme. It is useful in estimating infarction size in MI	(Anderon 2005)
IGF binding complex acid labile chain	Plasma	CAD	–	(Kontush 2016)
Insulin, insulin C-peptide	Plasma	Ischemic heart disease	In non-diabetic men, the higher plasma level of fasting insulin level is prone to ischemic heart disease	(J EAN -P IERRE D ESPRÉS, B ENOÎT L AMARCHE, P ASCALE M AURIÈGE, B ERNARD C ANTIN, G ILLES R. D AGENAIS, S ITAL M OORJANI 1996)
Insulin-like growth factor binding protein-7	Blood	Cardiomyopathy-induced heart failure	–	(Heald et al. 2001)
Insulin precursor	Plasma	CHD	Increased concentration of it predicts morbidity due to CHD	(Anderon 2005)
Intercellular adhesion molecule 1, soluble	Blood	Coronary heart disease	It estimates the risk of coronary heart disease	(Witte et al. 2003)
Interleukin-1 receptor antagonist	Serum	Ischemic stroke, MI, ACS and unstable angina	Increased level of IL-1Ra is found to be a biomarker for ischemic stroke, MI, ACS, and unstable angina	(Bonaterra et al. 2010) (Blake and Ridker 2003)
Interleukin-1 beta	Serum	MI	Its level is higher in MI	(Anderon 2005)
Interleukin-6	Serum	Inflammatory	It is an inflammatory biomarker. The higher level of IL-6 is associated with increased risk of cerebrovascular disease and cardiovascular disease	(Blake and Ridker 2003,2003)
Interleukin-18	Serum	ACS	Increased serum IL-18 value is found to be related to ACS, according to the clinical trials	(Bonaterra et al. 2010)(Moe and Wong 2010)
Interleukin-10	Serum	Stroke	Elevated interleukin-10 is detected in stroke patients	(Moe and Wong 2010)
Interleukin-2	Serum	MI	Its increased level is found in MI and UA	(Moe and Wong 2010)
Ischemia modified albumin	Serum	MI	IMA is released within minutes after the occurrence of ischemia. It stays elevated for 6–12 h and comes to normal level within 24 h. It is more sensitive than troponin in the diagnosis of myocardial ischemia	(Anwaruddin et al. 2005)
Isoprostanes	Urine	Atherosclerotic CVD	–	(Tsimikas 2006)
Lactate dehydrogenase	Serum	AMI	Other than the heart, LDH is expressed in organs like the liver, lung, erythrocytes, and kidney thus making it less specific	(Garg et al. 2017)(Danese and Montagnana 2016)
Lectin-like oxidized low-density lipoprotein receptor-1	Plasma	ACS	It is related to vascular inflammation as well as to atherosclerotic plaque. Plasma levels of LOX-1 are increased in the patients with ACS	(Johansson et al. 2018)
Leptin	Serum	CHF	Increased serum level of leptin is found in CHF	(Balagopal et al. 2011)
Lipoprotein-associated phospholipase A2	Blood	Vascular inflammatory and atherosclerosis	It is highly specific to vascular inflammatory and atherosclerosis than hs-CRP	(Lerman and McConnell 2008)
Microalbuminuria	Circulating	CHD and CVD	It predicts CHD and CVD. The variation in its level is observed based on age, gender, and status of diabetics	(Tehrani and Wong 2015)
Matrix metalloproteinase	Blood	CAD	They might be used as a marker for CAD, since it indicates plaque destabilization. Plasma levels of MMP-1, MMP-2, and MMP-9 are elevated in ACS patients	(Eckart et al. 2004)
Monocyte chemoattractant protein-1	Plasma	ACS and MI	The elevated plasma level of Monocyte chemoattractant protein-1 is linked with hyperlipidemia and MI. It is linked with coronary artery calcium levels	(Johansson et al. 2018)
Mucin cell surface associated protein 18 (muc18)	Plasma	MI	Plasma levels are found to be positively associated with MI	(Kontush 2016)
Multimerin-2	Plasma	MI	Plasma levels of multimerin-2 are found to be positively associated with MI	(Kontush 2016)
Myeloid-related protein 8/14	Circulating	ACS	Some studies have shown the elevation of the myeloid-related protein 8/14 complex in ACS	(Dekker et al. 2010)
Myeloperoxidase	Plasma	ACS	MPO is increasingly linked to ACS and monitoring the plasma levels of MPO may help in risk stratification of MI	(Morrow et al. 2008)
Myoglobin	Blood	MI	It clears from the blood rapidly; thus, it is not useful for patients who are presented late	(Brian Gibler et al. 1987)
Myosin light chain I	Serum	AMI	–	(Uji et al. 1991)
Myosin heavy chain	Plasma	Cardiac muscle damage	–	(Anderon 2005)
Myosin heavy chain 7	Plasma	HF	–	(Anderon 2005) (Kontush 2016)
Myosin light chain II	Plasma	Cardiac muscle damage	–	(Anderon 2005)
Neural cell adhesion molecule -1	Plasma	CAD	Decreased plasma level of NCAM-1 may be a marker for CAD	(Yu et al. 2018)
Neutrophil gelatinase-associated lipocalsin	Plasma	Stroke	The higher levels of Neutrophil gelatinase-associated lipocalsin is observed in stroke	(Anderon 2005)
Neutrophil protease-4	Plasma	Stroke	–	(Anderon 2005)
Osteoprotegerin	Blood	Cardiovascular mortality	–	(Browner et al. 2001)
Oxidized phospholipids	Blood	CAD	It is released in response to oxidative stress. It is bound to lipoproteins and is responsible for atherosclerosis	(Tsimikas 2006)
oxLDL	Plasma	CHD	Increased level of oxLDL is found in the plasma of CHD patients. Its association is also found in atherosclerotic disease, ACS, IMT, and plaque instability	(Bonaterra et al. 2010)
Paraoxonase	Plasma	Developing CVD	–	(Getz and Reardon 2004)
Plasminogen	Plasma	Thrombosis	–	(Anderon 2005)
Plasminogen activator inhibitor (PAI)-1 antigen	Plasma	Coronary artery disease and stroke	In coronary artery disease and stroke, increased plasma levels of plasminogen activator inhibitor (PAI)-1 antigen are observed	(Anderon 2005)
Plasminogen activator inhibitor-1	Plasma	Risk predictor for CVD	–	(Diamantopoulos et al. 2003)
Platelet- activating factor (PAF) acetylhydrolase	Plasma	MI, Stroke	Its deficiency is related to, MI, stroke, non-familial cardiomyopathy, and brain hemorrhage	(Anderon 2005)
Pregnancy- associated plasma protein-A	Serum	ACS	The elevated level of PAPP-A is found in unstable plaque and also in CAD. It can also indicate progression of the MI	(Laterza et al. 2004)
Protein C	Plasma	Hemostasis	It is a regulator of hemostasis	(Muscari et al. 1995)
Protein S	Plasma	Risk factor	–	(Muscari et al. 1995)
Prothrombin fragment 1. 2	Plasma	Stroke	Higher value is found in stroke patients than in controls	(Anderon 2005)
Quiescin Q6 (QSOX1)	Circulating	HF	–	(Kontush 2016)
Resistin	Plasma	CHD	The concentration of resistin helps in the determination of vasculature inflammation status, in turn the atherosclerosis progress	(Pischon 2009)
Retinol binding protein-4	Serum	MI	It is an adipokine. Lower level of retinol is found to be linked with MI	(H. J. Kim et al. 2011)
Salivary alpha amylase 1	Plasma	MI	Plasma levels of Salivary alpha-amylase 1 are found to be positively associated with MI	(Kontush 2016)
Secreted phosphoprotein 24	Circulating	CAD, HF	–	(Kontush 2016)
Secretory phospholipase A2	Plasma	Inflammation	–	(Koenig and Khuseyinova 2009)
Serum amyloid (SAA)	Serum	CVD and CAD	It is a marker for the inflammatory process. The increased level of CRP and SAA may predict the inflammatory process	(Tuñón et al. 2016)
Serum tartrate-resistant acid phosphatase isoform 5a	Serum	MI	–	(Janckila et al. 2011)
Soluble E-selectins	Plasma	Acute stage of ischemic	Increased level indicates acute stage of ischemic events	(Pletsch-borba et al. 2019)
Soluble intercellular Adhesion Molecule -1	Circulating	CAD	Increased level of circulating sICAM-1 is independently correlated to the CAD. Its association was also found in atherosclerosis	(Pletsch-borba et al. 2019)
Soluble P-selectins	Plasma	UA	Plasma levels of soluble P-selectins are found to be more than that of healthy individuals in patients with unstable angina, hypercholesterolemia, and hypertension	(Pletsch-borba et al. 2019)
Soluble tumor necrosis factor like weak inducer of apoptosis	Plasma	CAD and chronic heart failure	Lower level of sTWEAK is associated with CAD, systolic heart failure, atherosclerosis in chronic kidney disease, and peripheral artery disease	(Blanco-Colio et al. 2011)
Soluble vascular adhesion molecule-1	Serum	Atherosclerosis	The serum sVCAM-1 level may show the extent of atherosclerosis, thus may be used in the early stages. It is suggested to be the marker for endothelial dysfunction	(Bonaterra et al. 2010)
ST2	Serum	ACS and HF	The prediction of cardiovascular morbidity may be done with ST2 in patients with ACS. The increase in the serum sST2 value was found in HF patients	(Gruzdeva et al. 2019)
Surfactant protein D	Circulatory	Risk marker for CVD	Elevated level of surfactant protein in circulation may be a risk marker for CVD	(Hill et al. 2011)
Thrombin activatable fibrinolysis inhibitor	Circulating	Stability of clot	It affects indirectly the stability of the clot	(Anderon 2005)
Thrombomodulin	Plasma	MI and cardioembolic stroke	Lower level of thrombomodulin causes uncontrolled thrombus. Increased concentration is found in patients having MI and cardioembolic stroke	(Johansson et al. 2018) (Pletsch-borba et al. 2019)
Tissue factor	Plasma	MI	In patients with MI, higher levels of tissue factors are found	(Fareed et al. 1998)
Tissue factor pathway inhibitor	Plasma	AMI	Higher level is found in AMI	(Fareed et al. 1998)
Tissue inhibitor of metalloproteinases-1	Serum	MI and cardiac mortality	It is a predictor of MI and cardiac mortality	(Velagaleti et al. 2010)
Transforming growth factor-beta	Circulating	CAD	Lower concentration is found in patients with CAD	(Frishman 2002)
Tropomyosin	Serum	MI	It is elevated in patients with MI	(Cummins et al. 1981)
Troponin	Plasma	Cardiac damage	Troponin T can help to predict coronary disease even in the presence of renal dysfunction	(Bucciarelli-Ducci et al. 2004)
Tumor necrosis factor-alpha	Plasma	CAD	TNF-alpha is a marker for atherosclerosis. In premature CAD the increased level of plasma TNF-alpha is found	(Bonaterra et al. 2010) (Ruwanpathirana et al. 2015)
Tumor necrosis factor receptor I	Plasma	CVD mortality	It is an independent predictor of CVD mortality	(Anderon 2005)
Tumor necrosis factor receptor II, soluble	Plasma	CHF	Patients having CHF have a higher value of tumor necrosis factor receptor II, soluble	(Anderon 2005)
Type II secretory phospholipase A2	Circulating	CHD and atherosclerotic disease	The enzymes’ elevated activity shows its correlation with CAD. Its elevated levels are also observed in rheumatoid arthritis and sepsis	(Bonaterra et al. 2010)
Unbound free fatty acids	Serum	Acute myocardial infarction	It is a predictor of sudden death	(McDonnell et al. 2009)
Uric acid	Serum	Risk marker for CVD	Increased concentration of uric acid in serum is found to be an important risk marker for CVD	(Doehner and Landmesser 2011)
Vascular endothelial growth factor	Plasma	Peripheral artery disease	Peripheral artery disease patients are found with higher levels of vascular endothelial growth factor	(Makin et al. 2003)
Vinculin	Plasma	Atherosclerosis	Elevated plasma levels of vinculin are a predictor of atherosclerosis	(Kristensen et al. 2014)
Von Willebrand factor	Circulating	Cardiovascular risk factor	Higher concentration of vWF is associated with cardiovascular risk factors	(Frishman 2002)
Von Willebrand factor, propeptide	Plasma marker	Acute endothelial secretion	–	(Anderon 2005)
White blood cell count	Blood	CVD	It has a positive correlation with CVD	(D’Aiuto et al. 2013)
YKL-40	Serum	CAD and MI	YKL-40 is associated with endothelial dysfunction. It is an inflammatory glycoprotein. Variation in YKL-40 is seen in atherosclerosis. Higher concentration of YKL in serum is found to be associated with the extent of CAD and MI. Its value is higher in type 1 and 2 diabetes which is one of the risk factors of CVD. Elevated level of YKL is also found in diseases like cancer, rheumatoid arthritis, liver cirrhosis, and psoriasis	(Tan et al. 2019)

## Omic techniques for the detection of cardiac biomarkers

Biomarker detection in body fluids is an active area of research at present, due to its screening capability. The omic techniques (genomics, proteomics, metabolomics, breathomics, volatilomics, etc.) can be used for understanding the various molecular processes and understanding the correlation between the molecular species produced in an abnormal condition. The advancement in omic techniques helps in the analysis of complex samples with very little compromise on sensitivity and specificity (97) (Schneider and Orchard 2011) (X. Zhang et al. 2007). The omic techniques help in better understanding the pathophysiology of conditions like CVD and along with that provide a good platform for the detection of new biomarkers (Leon-mimila et al. 2019).

Genomics deals with the study of genes, in order to understand the role of genes in disease induction and progression. Some of the single-nucleotide polymorphisms are found to be related to coronary artery disease (CAD) (de Franciscis et al. 2016). The RNAs are studied in transcriptomics and messenger RNAs are the most investigated ones (de Franciscis et al. 2016) (Bank et al. 2015). The plasma concentrations of microRNAs (miRNAs) like miR-1, miR-133, miR-208a, and miR-499 are found to be increased in MI patients. Lower levels of miR-126 is found in CAD (de Franciscis et al. 2016). The role of miR-34 and miR-24 in the progression of MI has been studied by Zhen Wang et al. (Z. Wang et al. 2020a, b). Dongying Zhang et al. have studied the regulatory mechanisms of miR-519d-3p and Hox transcript antisense intergenic RNA (HOTAIR) in MI rats. From their experimental studies, they concluded that it can be used in MI diagnosis and therapy (Dongying Zhang et al. 2019). Pirouzpanah et al. have reported the diagnostic value of miRNA 21 for AMI (Pirouzpanah and Mohammad 2019). In patients with HF, the upregulation of miR5571-5p, miR-3135b, and miR3908 has been observed (F. Chen et al. 2018). Cosentino et al., in their study, observed the diagnostic and prognostic effect of mitochondrial biomarkers such as cytochrome C and cell-free mitochondrial DNA in STEMI patients. The mitochondrial biomarkers were found to be complementary to the troponins in acute ventricular dysfunction (Cosentino et al. 2021).

Small molecules (< 1500 Da) like peptides, amino acids, and carbohydrates are studied in metabolomics. Metabolomics can be used to identify various metabolites which are linked to CVD (Tuteja and Rader 2012). Amino acids like tyrosine, valine, phenylalanine and leucine are predicted to be associated with diabetes which is one of the risk factors of CVD. Higher concentrations of serum proteins, monounsaturated fatty acids, and phenylalanine along with reduced level of polyunsaturated fatty acids are found to be indicators of CVD (Tuñón et al. 2016). Christine et al., has studied the n-3 fatty acids and vitamin D supplementation effects in cardiac biomarker and inflammation in diabetes patients. The study concluded that the supplementation of vitamin D_3_ and n-3 fatty acid did not alter the concentration of inflammatory biomarkers in type-2 diabetes patients (Limonte et al. 2021).

Proteomics deals with (i) identification of proteins, (ii) protein profiling, (iii) determination of protein structures, (iv) protein–protein interaction, (v) protein quantification, and (vi) post-translation modification (Lindsey et al. 2015). Proteomics can provide details about the state of the cell, tissue, and organ (Lindsey et al. 2015). Even with the advancement of technologies, the sensitive detection of low abundant proteins, complex protein mixture analysis, quantification of protein, etc. still remains a challenging task. Thus, detection of protein biomarkers is still not easy due to the very large numbers of proteins in samples (blood serum, tissue, urine, etc.), a wide range of protein concentrations, etc. (Chandramouli and Qian 2009)(Pirouzpanah and Mohammad 2019). The posttranslational modifications (PTM) are significantly involved in the CVD process but are not easily studied by using genomics and transcriptomics (Shen et al. 2014). Studies involving the phosphorylation of troponin have been carried out in ischemic heart patients to healthy donors, illustrating the importance of PTMs (Shen et al. 2014). Proteomics studies have also been carried out after reducing the complexity of the samples by extracting the high abundant proteins like albumin, IgG, IgA, transferrin, haptoglobin, and antitrypsin (Yu et al. 2018) (H. J. Kim et al. 2011).

## Spectroscopic techniques for cardiac marker detection

### Surface-enhanced Raman spectroscopy

Spectroscopic techniques have been gaining attention in cardiac diagnostic applications due to their potential for rapid detection of biomarkers, with very high sensitivity. Fluorescence, surface-enhanced Raman spectroscopy (SERS), photoacoustic absorption, and surface plasmon resonance (SPR) have been always at the forefront of clinical spectroscopy due to the enormous possibility of translating into point-of-care (POC) devices. These techniques have been highly beneficial for biomarker detection due to their rapid, label-free detection with minimal or no sample processing protocol. Raman spectroscopy is a vibrational spectroscopic tool which can provide information regarding the biological entities and their environment by evaluating the inelastically scattered light properties originating from samples upon laser excitation. The low sensitivity of the conventional Raman spectroscopic technique which has got lower detection limits can be overcome by the use of metallic nanoparticles or rough, specially designed, metallic substrates in what is termed surface-enhanced Raman spectroscopy. The combination of electromagnetic field enhancement and charge transfer mechanism involved in this technique can provide for the Raman signal enhancement up to 10^14^ or more, even down to a single molecule (Langer et al. 2019) (Kneipp et al. 1998)(John et al. 2022).

Chon et al. have introduced a rapid, sensitive approach for the simultaneous detection of cardiac markers, troponin, and creatine kinase using SERS immunoassay measurements. This competitive immunoassay employing the combination of SERS nanotags and magnetic beads was effective in the detection of 2.5 and 33.7 pg/mL for creatinine kinase and troponin respectively (Choo 2014). This methodology was claimed to be less influenced by sample matrix effects and also does not demand any pre-processing approaches like filtration and centrifugation. Another rapid, cost-effective SERS immunoassay approach developed by Su et al. has been capable of simultaneous detection of three biomarkers (cardiac troponin I (cTnI), N-terminal prohormone of brain natriuretic peptide (NT-ProBNP), and neutrophil gelatinase-associated lipocalin) with minimal sample volume (Su et al. 2018). Further, they have obtained a LOD of 0.76, 0.53, and 0.41 fg/mL for cTnI, NT-ProBNP, and NGAL, respectively, and later, the assay was validated in the blood plasma collected from the patients, which affirms the potential of this technique for POC clinical diagnostics (Su et al. 2018). Another interesting SERS lateral immunoassay introduced by Fu et al. has explored the use of graphene oxide–gold nanoparticle complex for efficient signal amplification for troponin detection. This assay was able to perform quantitative detection of troponin at a significantly lower range (5 pg/mL to 1000 ng/mL) which was otherwise not in the achievable limit of immunoassay without graphene oxide (Xiuli et al. 2018). In order to circumvent the limitations with the measurement of SERS nanoprobes in biological solutions, Garza et al. have designed and developed a novel SERS collection device which can consistently gather the nanoprobes for SERS measurement. The precipitation of nanoprobes created in solutions can move away from the laser excitation spot during the usual SERS measurements, which results in smaller number of sample molecules, affecting sensitivity. The sensing approach by Garza et. al. demonstrated an enhancement in SERS signal intensity of cardiac troponin with respect to the traditional nanoprobes in solution approach. Researchers have also encoded SERS nanotags with lateral flow assay immunostrips to generate ultrasensitive POC, multiplex detection of cardiac markers (Garza and Cote 2017) (Di Zhang et al. 2018). The use of SERS encoded with lateral flow assay (LFA) strips with one test line has resulted in the reduction in cost, complexity of operation, sample volume, and reagent consumption (Di Zhang et al. 2018). S. Mabbot et. al. have fabricated a paper-based microfluidic assay for the detection of miR-29a, a micro-RNA (miRNA) biomarker of CVD (Mabbott et al. 2019). The device comprising dual analytical readouts (colorimetric and SERS) was proposed to be a viable option for point-of-care detection of miRNA circulating freely in the bloodstream. An LOD of 47 pg /μL has been obtained using this SERS test. Aptamer-based SERS biosensing platforms have been also reported capable of detecting cTnI as low as 10 ng/ml. A. Waleed et. al. have reported myoglobin detection in buffer and urine samples by constructing a 3D silver anisotropic nano-pine tree array-modified indium tin oxide SERS substrate (El-said et al. 2016). Benford et al. have developed a SERS-based biosensor consisting of a nanofluidic channel for the detection of multiple analytes (brain natriuretic peptide, troponin I, and C-reactive protein). SERS hot spots in the measurement region of the nanofluidic channel were generated with the help of high-density aggregated gold nanoparticles ~ 60 nm in size (Benford et al. 2009). A recent SERS assay work have employed gold patterned array chip and core–shell nanoparticles for the ultra-sensitive duplex detection of cardiac troponin I (cTnI) and creatine kinase-MB from serum samples. The developed assay method has the detection limit for cTnI and creatine kinase-MB as 8.9 pg/mL and 9.7 pg/mL respectively, which is highly sensitive than the traditional fluorescence or ELISA approaches. (Cheng et al. 2019). The SERS technique has been also employed for the quantitative detection of heart-type fatty acid-binding protein (Ma et al. 2019). This technique which obtained a LOD ~ 1.4490 ng/mL was better than the conventional ELISA and colloidal gold immunochromatography techniques in terms of sensitivity, time consumption, and ease of operation. M. Shorie et al. have developed gold nanoparticle–tungsten disulfide SERS substrate which can facilitate combined effects of chemical and electromagnetic properties for multifold Raman signal enhancement of probe molecules (Shorie et al. 2018). The nanohydrid (Gold nanoparticles (AuNPs) are assembled on exfoliated tungsten disulfide nanosheets (WS2)) substrate has been effective in obtaining a detection limit ~ 10^−2^ pg/mL for myoglobin, which is sufficient enough for clinical applications. A recent SERS work using Au core–Ag shell nanotags has obtained a detection limit of 9.80 pg/mL for troponin with high specificity. The detection, carried out using a portable Raman device, was fast, with minimal sample preparation and low sample volume (50 μL) and has been suggested as a reliable POC method in clinics for early detection of cardiac disorders (Shorie et al. 2018). The same research group have also used the core–shell-mediated substrate for simultaneous detection of the Heart-type fatty acid-binding protein (H-FABP) and cardiac troponin I with LOD of 0.6396 and 0.0044 ng /mL respectively (Hu 2020).

#### Surface plasmon resonance

**Fig. 1:**
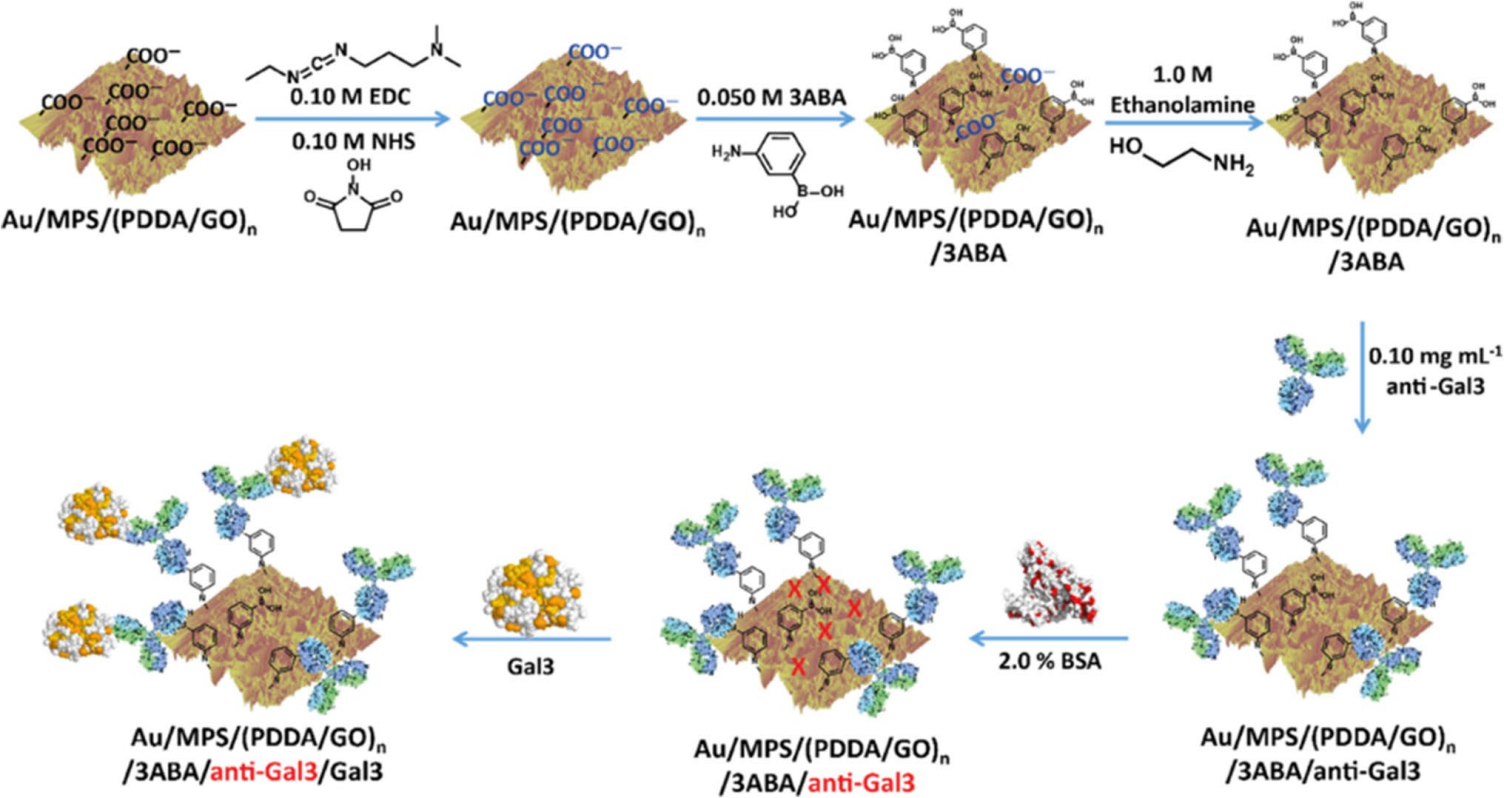
The Gal3 immunosensor has been built using the Au/MPS/(PDDA/GO)n platform, as shown in this schematic (Primo et al. 2018) [Reproduced with permission]

The surface plasmon resonance technique basically relies on collective oscillations of surface electrons at a metal–dielectric interface, induced by light. Such metallic structures with a highly localized electromagnetic field have got high sensitivity to any changes occurring to the refractive index in the medium in contact with it. This property can be widely explored for various biosensing purposes, and thus, the SPR technique has found ubiquitous use in the detection of pathogens, biomarkers, toxins, allergens, etc. (J Lukose et al. 2016)(Jijo Lukose et al. 2018). Primo et. al have performed SPR measurements using the commercial platform Autolab SPRINGLE for the detection of galectin-3 (Gal3) with an LOD of ~ 2.0 ng /mL, a promising marker for cardiac diagnosis (Primo et al. 2018). This immunosensing technique was developed by fabricating a self-assembled layer of poly-(diallyldimethylammonium chloride) and graphene oxide (GO) on gold surface and covalently linking an orientation element (3-aminephenylboronic acid) for anti-Gal3 attachment. The use of graphene oxide has a significant impact on enhancing the sensing performance whereas the orientation element resulted in increased specificity for the anti-Gal3 towards Gal3 binding. Au/sodium 3-mercapto-1-propane- sulfonate (MPS) was made to interact with a PDDA solution for about 15 min. Then Au/MPS/PDDA solution was made to interact with a GO solution for 30 min. The multilayer system (Au/MPS/(PDDA/GO)_*n*_) was obtained by repeating the procedure n times. EDC/NHS mixture was used to activate the GO carboxyl residue and left to interact with 3-aminephenylboronic acid (3ABA) solution for amidation reaction. The non-reacted residue was quenched in ethanolamine (EtNH2) solution. A BSA solution was used in Au/MPS/(PDDA/GO)n/3ABA/anti-Gal3, for blocking the non-absorbing sites, and the procedure is described in Fig. 1. This SPR technique has also displayed higher selectivity while performing experiments in human serum samples (Primo et al. 2018). Jang et al. have utilized nanoparticle-enhanced SPR methodology for the detection of marker protein B-type natriuretic peptide (BNP), in the range 1 aM to 500 nM (Jang et al. 2014). This sandwich immunoassay was fabricated using aptamer functionalized metallic surface in combination with gold nano-cubes attached with antibody and carried out measurements successfully in buffer and undiluted serum sample. Liu et al. have carried out troponin detection using an SPR biosensor with anti-fouling ability for specific attachment of target biomarkers (Tsai et al. 2011). Even though the detection was carried out in less than 2 min, the detection limit (100 ng/mL) obtained was not sufficient enough to be used for measurements with clinical samples. Troponin measurements using commercial SPR platforms (K-MAC micro SPR model, Korea and (AutoLab Spirit®, Eco Chemie, The Netherlands)) have also obtained comparable performance with that of ELISA assays (Y. Kim and Kwon 2011) (Fireman and Tatsuo 2007).

**Fig. 2:**
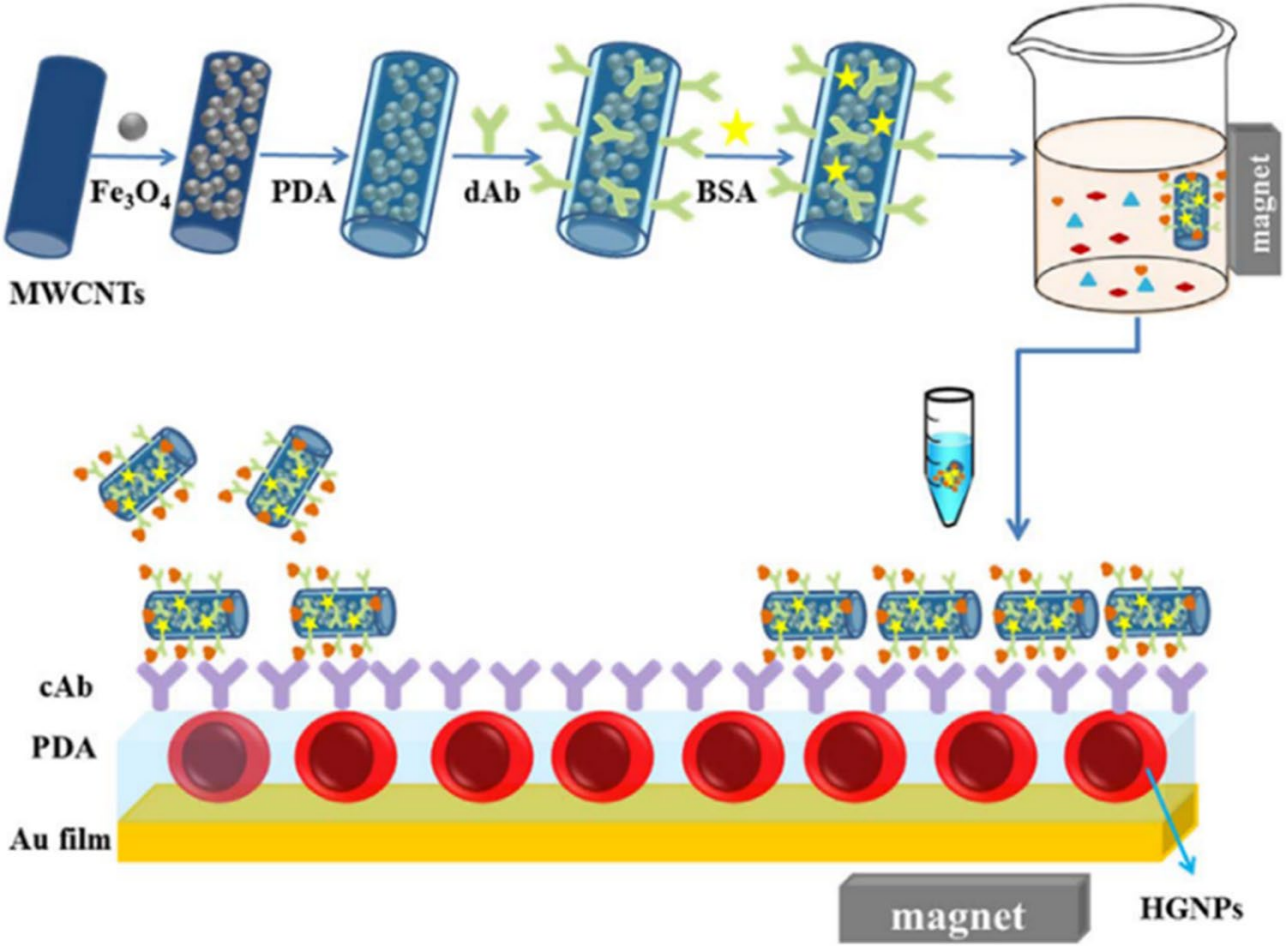
Schematic diagram SPR biosensor based on MMWCNTs-PDA immune probe for the detection of cTnI (Wu et al. 2017). [reproduced with permission]

Wu et al. have achieved ultrasensitive troponin detection using magnetic field–assisted SPR immunoassay approach (Fig. 2). The technique involving a magnetic immune probe has obtained 1.25 ng/mL detection limit for troponin, which was ~ 1000-fold better than the traditional polydopamine-based SPR assays (Wu et al. 2017). Kurita et al. have reported trace level detection of BNP peptide (15 fg) in 30 min. time by using a combined microfluidics-SPR approach (Kurita et al. 2006). Masson et al. have fabricated a SPR-based fiber optic probe for myoglobin and cardiac troponin I sensing within ten minutes time and detection limits of 1.4 and 2.9 ng/ml respectively (Masson et al. 2004). Tadepilli et al. have fabricated plasmonic paper based sensor capable of detecting troponin I in human serum and sweat samples (Tadepalli et al. 2015). The short peptide based plasmonic sensors are suggested as reliable point of care sensors, due to their high thermal stability, longer shelf life and high sensitivity. Similarly, LSPR based detection of troponin in human serum was also performed by Ding et al. using nanoimprinted, large surface area, nanohole array biosensor chips (Ding et al. 2015). The sensor showed high reproducibility in measurements and have also obtained an LOD of 0.55 ng/ml. Liyanage et al. have fabricated gold nano-prisms functionalized with anti-cTnT for the detection of troponin with atto-molar detection limit (Liyanage and Andeep 2017). Magnetic nanoparticles due to their high refractive index and high molecular weight can enhance the LSPR response of metallic nanoparticles for biosensing (Tang et al. 2013). Tang et al. have demonstrated this by using Fe_3_O_4_ magnetic nanoparticles along with gold nano-rods for troponin sensing from blood plasma (Tang et al. 2013). This resulted in a significant increase in the LSPR shift upon troponin binding (~ sixfold) followed by a detection limit down to picomolar level. In his previous work, Tang et al. have used a mixture of gold nanorods with tunable plasmon band for the simultaneous detection of troponin and myoglobin from sample solution (Tang and Casas 2014). The use of gold nanorods have been found beneficial for plasmonic sensing due to their ease of synthesis and the ability for creating tunable resonance band by varying the size and aspect ratio during synthesis. In addition, the background noise of endogenous chromophores from biological mixtures (e.g., serum and blood) is minimal in the wavelength range used. Huang et al. have incorporated LSPR approach integrated into a four channel microfluidic biosensing device for the multiplex detection of inflammatory biomarkers from ~ 60 μL of assay volume (J.-S. Chen 2020). The proof-of-concept model demonstrated IgG, CRP, TNF-α, and TNF-α/IgG multiplex detection in 3.5-h time. In a recent work, SPR technique has been coupled with mass spectrometry for the detection of myoglobin, the marker protein for heart muscle injury (J.-S. Chen 2020).

### Breathomics

Like the “Marker Proteins” produced in abnormal conditions, the bio-molecular reactions taking place during the induction, progression, regression, and recurrence of any disease, produce many volatile molecular species, mainly “volatile organic compounds, VOCs”. These will be carried over to the lungs through the circulating blood and eventually will come out through the exhaled breath. Breath analysis, called “Brethomics” or “Volatolomics,” thus offers a very convenient method for screening, early detection, and follow-up in many diseases, including cardiovascular conditions (V. R. et al. 2021)(Kartha and Santhosh 2014)(Bykova et al. 2019)(Marcondes-Braga et al. 2016)(Ibrahim et al. 2019).

**Fig. 3:**
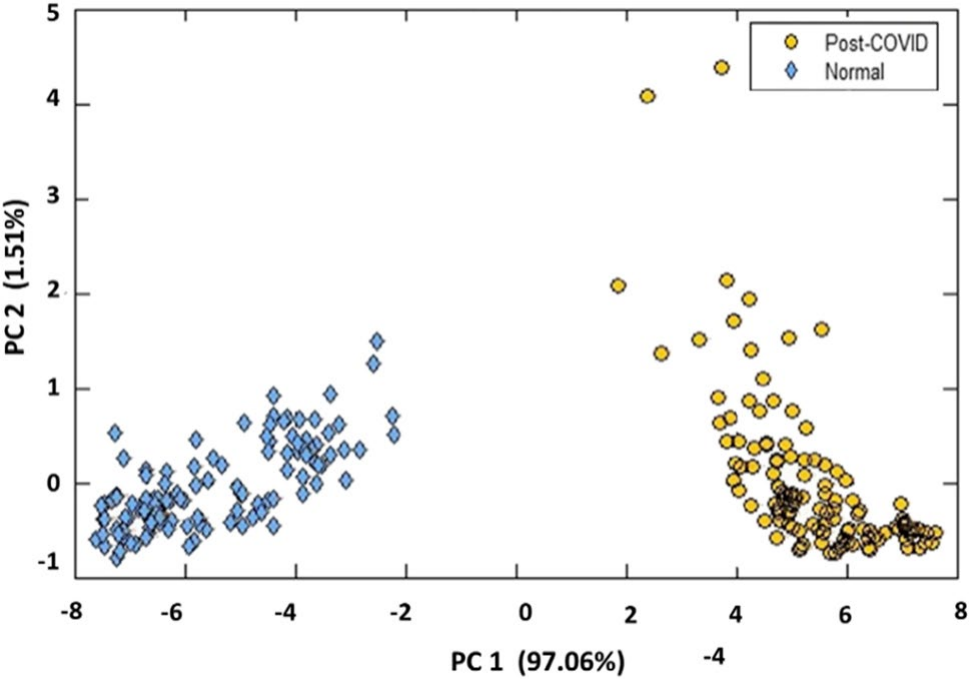
Score plot (PC1 vs. PC2) in PCA space with autoscale obtained from k-NN analysis of PCS and normal breath samples. (VR et al. 2022)

Breath analysis has thus attracted considerable interest recently, especially in view of the widespread pandemic condition of COVID-19, which often leads to many effects like Post-Covid Syndrome-PCS and Multiple Inflammatory Syndrome-Children, MIS-C, and several groups are carrying out vigorous research in developing breath analysis methods for diagnosis of cardiovascular diseases associated with such conditions. A typical application of the breath analysis technique in discriminating PCS-Post Covid Syndrome, from other conditions showing similar symptoms has been developed recently (VR et al. 2022). Figure 3 shows the classification obtained for PCS and normal breath samples using E-nose. It is clear that the breath biomarker associated with the PCS condition makes this classification in the score plot.

**Fig. 4:**
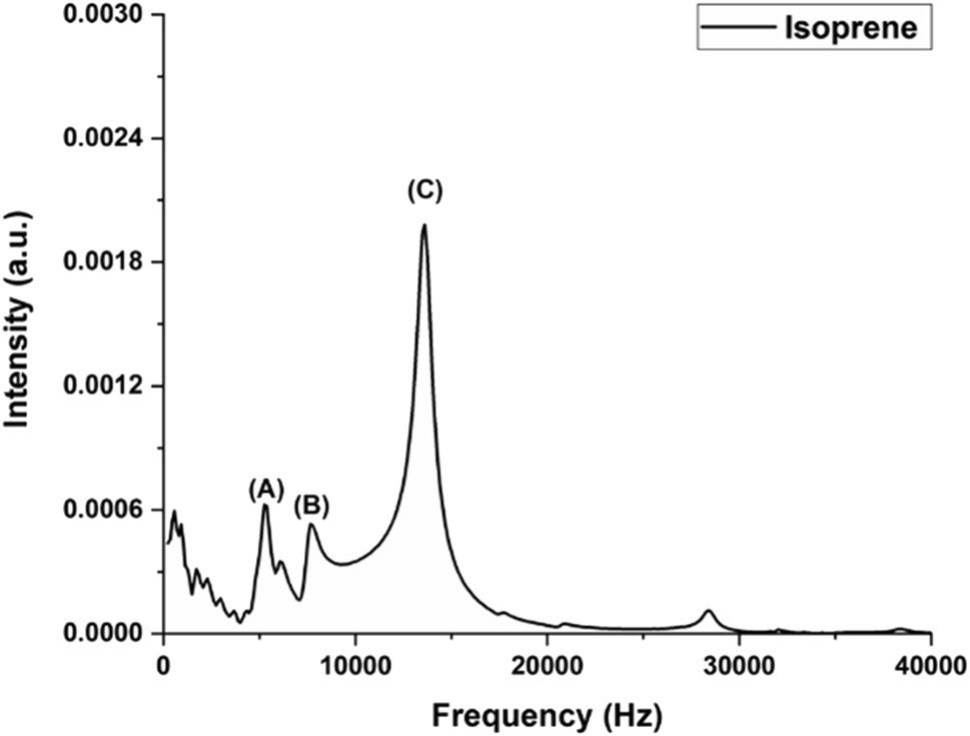
PA signal of isoprene vapor (98 ppb) obtained using 266-nm excitation. **A**, **B**, and **C** are the acoustic modes of the PA cell

The main methods used for breath analysis include spectroscopic, electronic, and mass spectroscopy-coupled techniques like gas chromatography-mass spectroscopy, GC–MS, and differential mass spectrometry. Several molecules like NO, acetone, isoprene, trimethyl amine, pentane, CO, ethane, etc. have been found to be good markers for many CVD conditions (Bykova et al. 2019)(Marcondes-Braga et al. 2016) (Cikach Jr and Dweik 2012). Table 2 shows the major breath markers observed in cardiovascular diseases. Many of these molecules are indicators of diseases which may later be involved in the development of cardiac conditions and periodic monitoring of them in the breath sample can thus serve as an efficient technique for early detection of CVD. For example, acetone, which is an indicator of diabetes condition, can be easily detected by UV-PAS (Nidheesh et al. 2021). Similar studies of breath constituents like isoprene, using PAS have shown their ability for Breath analysis, where even sub-ppb levels of these molecules can be detected by the PAS method (Fig. 4), this study can be further extended for the diagnosis/monitoring of cardiac conditions.

**Table 2:** Breath markers in cardiovascular diseases

Compound	Potential source	Implications for disease	Technology	Reference
Isoprene	Cholesterol synthesis	CVD	GC–MS, SIFT-MS	(Cikach Jr and Dweik 2012)
Acetone, isoprene, pentane, and ethane	Lipid metabolism Cholesterol biosynthesis Lipid peroxidation	Cardiometabolic disease	GC	(Owlstone 2022)
Pentane	Lipid peroxidation	Acute cardiac allograft rejection	GC	(Sobotka et al. 1994)
Acetone		Heart failure diagnosis	E-nose	(Yokokawa et al. 2016)
C_5_H_12_, N_2_O, NO_2_, C_2_H_4_, CO, CO_2_		AMI	Laser photoacoustic spectroscopy	(Borisov et al. 2021)

### Other methods

Blanco-Colio et al. identified soluble tumor necrosis factor-like weak inducer of apoptosis (sTWEAK)—a new biomarker for CVD through SELDI-TOF MS (Blanco-Colio et al. 2011). Madeleine Johansson et al. studied the relationship between orthostatic hypotension (OH) to that of CVD. Blood samples were analyzed by antibody-based proximity extension assay technique, combined with Olink Proteomics Proseek Multiplex CVD I 96 × 96 reagents kit through which they measured 92 CVD-related protein biomarkers. They identified proteins like MMP-7, TM, MB, TIM-1, CASP-8, CXCL-1, Dkk-1, LOX-1, MCP-1, PAR-1, PIGF, and TF, associated with atherosclerosis, MI, etc. (Johansson et al. 2018).

Capillary electrophoresis in combination with mass spectrometry (CE-MS), which was used for the peptide profiling of urine sample, is a reproducible technique. Five collagen fragments were identified through capillary electrophoresis coupled to micro-TOF mass spectrometry in patients with coronary artery disease (Dawson et al. 2012). It has high resolution but low sample loading capacity. Another study gave a panel of 17 peptides identified through CE-MS with some of them being collagen fragments. The same research team has done the study with 586 urine samples using CE-MS and identified 238 discriminatory polypeptides among which were fragments of alpha-1-antitrypsin, collagen types 1 and 3, granin-like neuroendocrine peptide precursor, membrane-associated progesterone receptor component1, sodium/potassium-transporting ATPase gamma chain, and fibrinogen-alpha chain (Delles et al. 2010).

Monocyte antigen CD14 has been identified through one-dimensional SDS-gel electrophoresis followed by liquid chromatography coupled with tandem mass spectrometry (LC–MS/MS) in patients with CAD. The validation of the same was done by ELISA in urine and serum samples (Lee et al. 2015). The levels of neutrophil gelatinase-associated lipocalin (NGAL) which is expressed in endothelial cells and modulate the activity of matrix metalloproteinase 9 (MMP9) and also an important mediator of vascular remodeling and plaque instability in atherosclerosis. C57BL/6 J control mice and atherosclerotic apolipoprotein E (apoE) low-density lipoprotein receptor (LDLR) mice were anesthetized and were given a brief hypoxic stress (10 min of 10% oxygen). Mice were allowed to come to a normal environment in 48 h. The expression of NGAL and MMP 9 was measured in the mouse 48 h later quantitatively through RT-PCR, zymography, and immunohistochemistry. The presence of NGAL and MMP9 in vascular inflammation caused by MI was suggested by the study (Hemdahl et al. 2006).

**Fig. 5:**
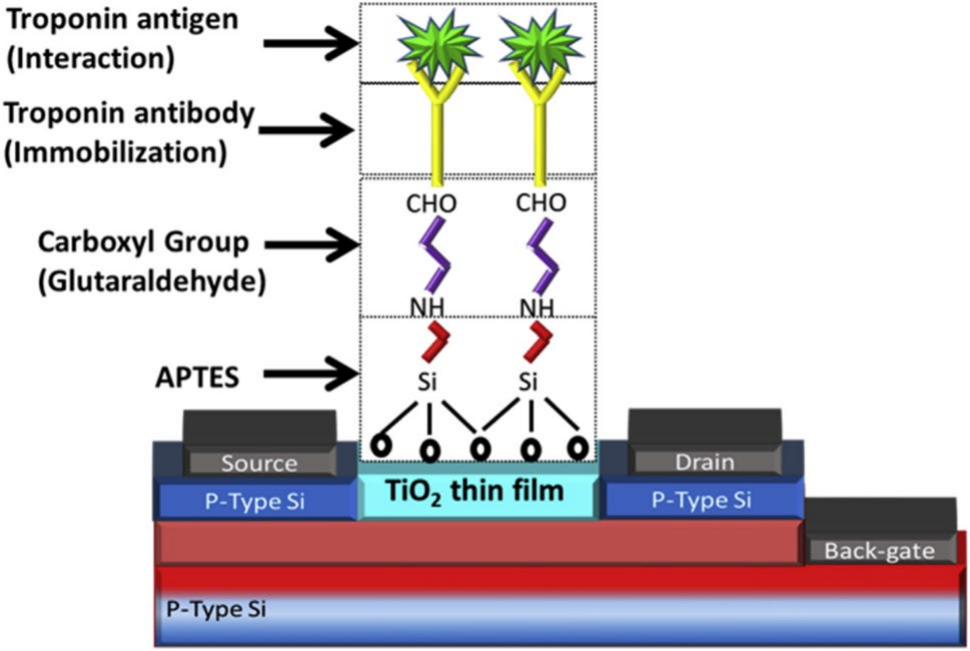
Covalently bonded functional groups which are involved in the surface immobilization process are illustrated in the diagram (Adzhri et al. 2017) [reproduced with permission]

Biosensors have also been designed to detect and quantify target molecules like proteins and nucleic acids or monitor antigen–antibody interaction. A fabrication procedure generally involves immobilization of DNA, RNA, antibody, etc. on the transducer surface, which converts the interaction between the target molecules and biological elements to a quantifiable signal. Optical biosensors work based on changes in amplitude, polarization, and frequency of input light or phase change in response to the biorecognition process (Qureshi et al. 2012). In the development of optical biosensors, the selection of a proper immobilization strategy plays an important role. An immobilization strategy includes enhancing the surface area so that the biological element of interest securely fixes onto the surface, and the bioreceptor is optimally positioned (Regan et al. 2018). In one technique, p-type anatase was integrated with FET for the detection of troponin I. Demonstration of the device was done to detect the concentration of antigen troponin I in the range of 1 ng/ml to 10 μg/ml. Figure 5 depicts the FET-based immunosensor for troponin I detection (Adzhri et al. 2017). Voltamo-metric biosensor has been developed by Pourali et al., for the detection of troponin in AMI (Pourali et al. 2021). Jing Li et al. developed a silica-conjugated microcomb electrode sensor for the detection of troponin I which has the LOD of 1fM(Li et al. 2021). An aptamer-based plasmon enhanced electrochemiluminescence setup was developed for the detection of troponin I, by Kitte et al., the obtained LOD was 0.75 fg/mL (Addisu et al. 2021). Optical biosensors such as colorimetric luminescence and fluorescence biosensors use target molecules tagged with dyes which require expertise for their proper use; these biosensors are sensitive but are expensive and bulky (Qureshi et al. 2012).

Natarajan et al. developed a cellulose paper–based lateral flow immunoassay for the detection of troponin. The analytical strip is made of commercially available filter paper. The detection limit was 2.10–2.75 ng/ml (Natarajan, Jayaraj, and Prazeres 2021). Philps has developed a POC sensor, to detect and quantify the cardiac biomarker (Troponin, BNP), called Minicare. The device is based on sandwich immunoassay using magnetic beads, and the Minicare device uses frustrated total internal reflection (f-TIR) for the quantification of biomarkers. The device has a limit of detection of 18 ng/L for troponin T (156).

Fluorescence is a popular optical method for the detection and quantification of biomarkers. The specificity of the method is amplified by using labeling antibodies. The AQT90 FLEX system, developed by the Radiometer company, uses time-resolved fluorescence and immunoassay cartridge, to detect cardiac biomarkers like troponin and BNP (156). The device pylon developed by ET Healthcare also uses fluorescence for the detection and quantification of biomarkers. The device has a LOD of 1.2 ng/L for troponin (Buhot 2020).

Being an analytical tool, Fourier transform infrared (FTIR) spectroscopy has also gained high attention due to its enormous potential in investigating all kinds of samples comprising both chemical and biological species (Sala et al. 2020). In conventional FTIR spectroscopy, the IR beam will be directly transmitting over the sample of interest, whereas the variant Attenuated Total Reflection (ATR) mode (ATR) involves the monitoring of the variations in the IR radiations, once it is reflected from the sample which is kept on the top of an ATR crystal. Haas et al. have explored the potential of FTIR spectroscopy for diagnosing myocardial infarction from minimal amount of dried serum samples (Haas et al. 2010). The study comprising of the Raman data obtained from 225 healthy subjects and 342 cardiac subjects were subjected to both cluster analysis and artificial neural networking (ANN). The sensitivity and specificity for the myocardial infarcted vs healthy subjects were obtained as 98% and 97% respectively in ANN analysis. In a similar manner, 100% sensitivity and specificity was obtained for the discrimination of heart failure and myocardial infarcted classes. In another interesting work, the ATR-FTIR technique was employed for the quantitative investigation of choline from blood serum samples (Khanmohammadi et al. 2015). The elevated levels of choline in the blood of acute coronary syndrome patients have generated high interest among scientists to probe the choline concentration. The normal range of choline ~ 7–12.3 mmol/L in blood plasma will be increased significantly above 25 mmol/L for cardiac patients (Danne et al. 2007). The ATR study combined with various chemometrics tools have shown to be effective on the basis of studies performed with 82 samples. Researchers have also verified the utility of the FTIR technique for probing tissue samples in view of investigating cardiac disorders. R. Cheheltani et al. have reported FTIR imaging spectroscopy, which can assess collagen deposition in heart tissue as a result of myocardial infarction (Cheheltani et al. 2012). The study conducted on the tissues collected from rat models was effective at probing the IR characteristic band of collagen at 1338 cm^−1^. In another study, Zheng et al. have investigated paraffin-embedded heart samples for protein characterizing in the myocardial infarcted cases, where an enhancement in the α-helix and deduction in β-sheet of protein secondary structures were found with respect to control (Zheng et al. 2010). FTIR analysis on human atherosclerotic plaques is also reported in view of studying atherosclerosis, in which the predominance of spectral features arising from lipids, esters, fibrous tissues, and phosphate were found (Dritsa 2012). Table 3 summarizes various advantage and disadvantage of various spectroscopic techniques.

**Table 3:** Advantage and disadvantage of various spectroscopy techniques

Technique	Advantage	Disadvantage
SERS	Highly sensitive, label-free detection with minimal or no sample processing protocol	Substrate may degrade with time. Poor reproducibility
SPR	Label free, small sample size	Reusability of gold chips is a concern
Mass spectroscopy	Highly sensitive	Labor intensive. Sample processing is complex
Capillary electrophoresis	Low sample volume. Easy to operate	Less sensitive
HPLC-LIF	Sensitive. Easy to operate. Minimum sample volume	Not suitable for identification
Breath Analysis	Noninvasive, short analysis time, easy to operate	Selection of VOCs for particular application is difficult

### High-performance liquid chromatography-laser-induced fluorescence

**Fig. 6:**
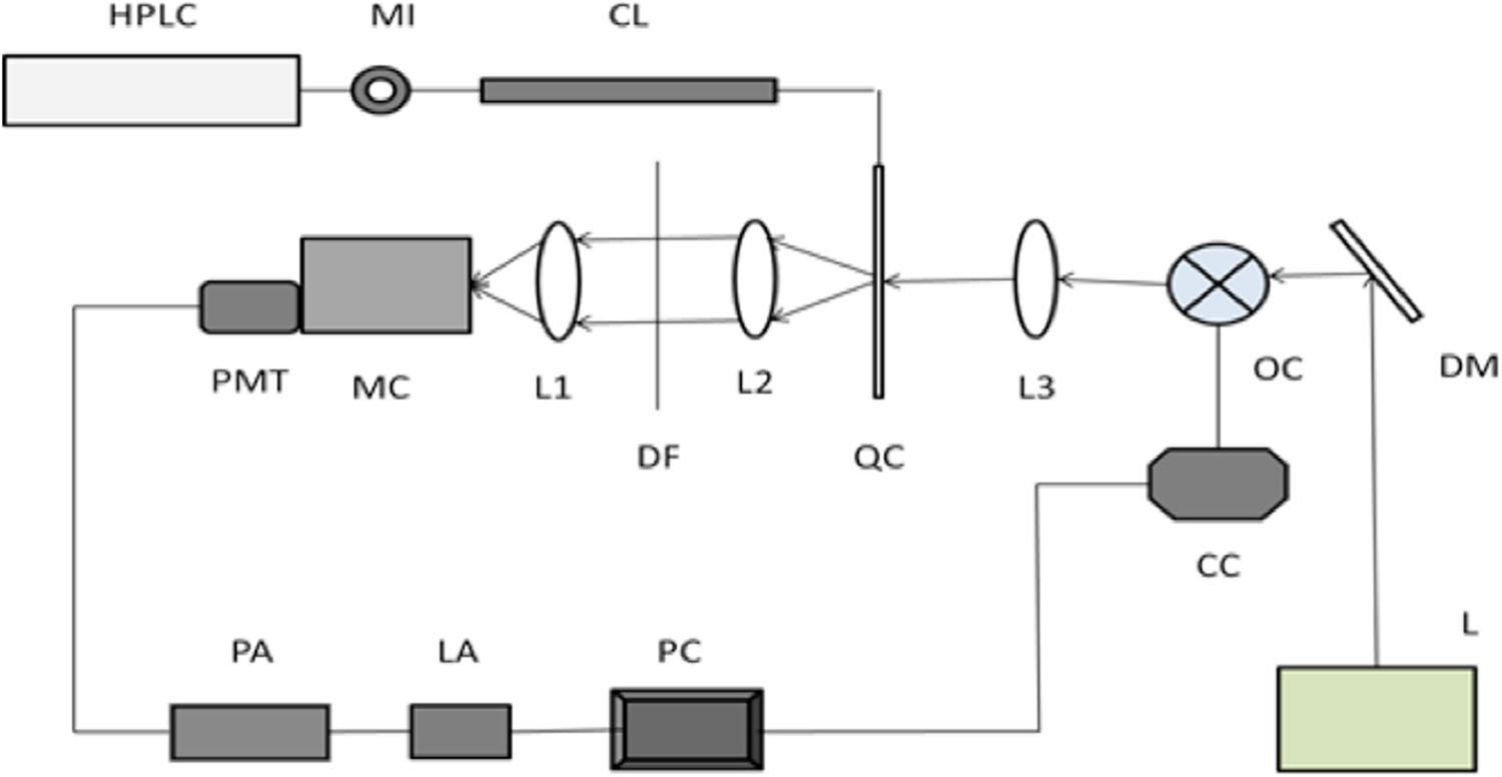
Experimental setup for HPLC-LIF system: MI, manual injector; CL, column; PMT, photomultiplier tube; MC, monochromator; L1, L2, L3, lenses; DF, dichroic filter; QC, quartz capillary; OC, optical chopper; DM, dichroic mirror; CC, chopper controller; PA, preamplifier; LA, lock-in amplifier; PC, computer; L, laser

We have assembled and used an ultra-sensitive high-performance liquid chromatography-laser induced fluorescence detection (HPLC-LIF) system in a protein-profiling approach for analyzing protein profiles of microliter quantities of clinical samples, such as serum, saliva, lysed cellular samples, and tissue homogenates (Venkatakrishna et al. 2003). The method is highly objective and capable of discriminating the sample under investigation as normal, pre-malignant, or malignant condition and malignancy stages. The system records the protein profile of clinical samples using a HPLC protein separation step combined with an ultra-sensitive laser-induced fluorescence (LIF) detection which provides the chromatogram, a plot of fluorescence intensity of the eluted protein versus time of elution (Bhat et al. 2010). The system can detect sub-femto-mole levels of proteins using microliter/gram amounts of a clinical sample and can give more or less complete profiles of the large number of proteins present at ultra-trace levels in these samples. Figure 6 depicts the schematic diagram of the setup. LASER (257 nm) is made to focus on the capillary flow cell through which the separated components from HPLC is made to pass. The fluorescence signal from the excited component is collected using collection optics along with a monochromator and detected using photomultiplier tube (PMT) (Bhat et al. 2010).

**Fig. 7:**
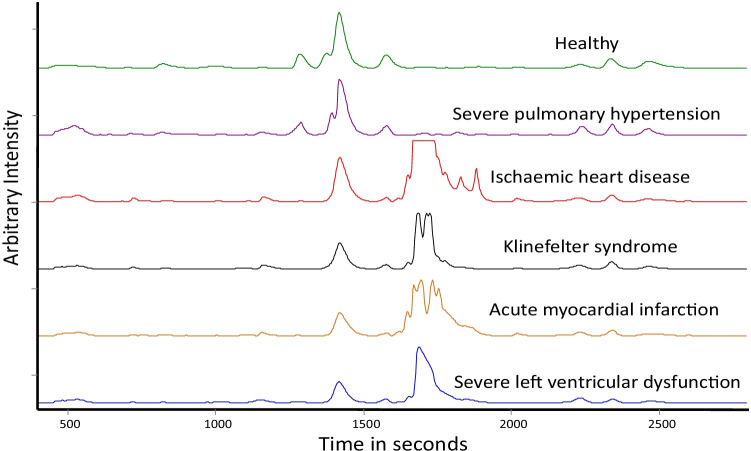
Typical serum protein profiles of healthy and various cardiovascular disease conditions

**Fig. 8:**
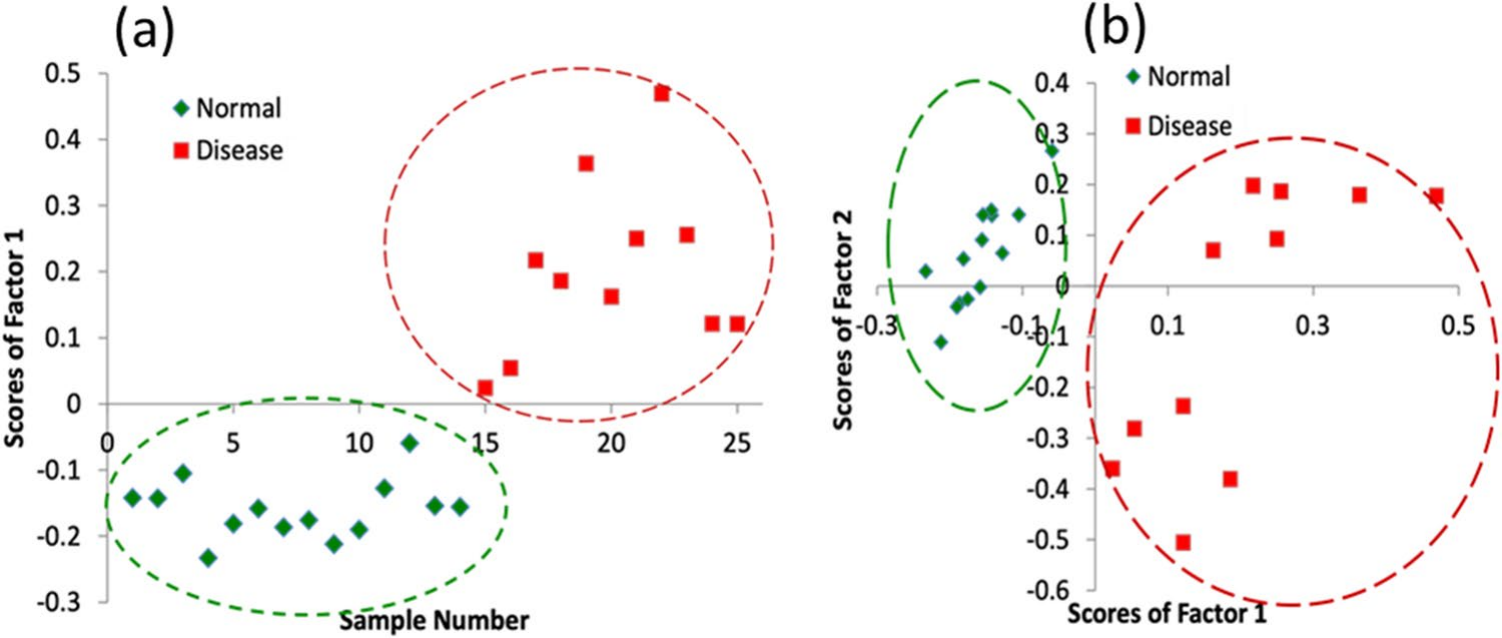
Principal component analysis results. **a** Scores of factor 1 vs. sample number and **b** sample scores of factor 1 versus scores of factor 2 (14 healthy and 11 CVD)

We have extended recently, our ultra-sensitive HPLC-LIF technique for the analysis of blood serum of CVD subjects (Rao et al. 2020). Serum protein profiles of healthy volunteers (14) and patients with different heart diseases (11) were recorded, using the HPLC-LIF system developed in our laboratory. Protein profiles of disease conditions can be seen in Fig. 7 which shows that there are significant differences in the protein peak positions and relative intensities, showing that the relative concentrations of many proteins present in normal and disease conditions are different, and that many new proteins are also formed in induction and progression of the disease. Preliminary data analysis (principal component analysis, PCA) on recorded protein profiles has given good discrimination between protein profiles of healthy subjects and patients with different heart diseases (Fig. 8a and b). Further, a detailed protein profile study using a highly sensitive HPLC-LIF system is being carried out for detecting specific markers of various cardiovascular diseases.

In our recent work, we have extended the ultra-sensitive HPLC-LIF method for the analysis of serum protein profiles of ACS samples. The blood samples (45–70 years) are collected from the patient admitted in the cardiology ICU/ward with diagnosis of ACS (ECG changes/elevated cardiac biomarker) department of cardiology, Kasturba Medical College, Manipal. Normal blood samples were collected from age matched healthy volunteers. The ethical clearance has been obtained for the study, from Institutional Ethics Committee. All samples were used with “informed consent.” A total of 17 normal samples and 32 ACS samples were collected. Experimental details have been explained elsewhere (Rao et al. 2020)(Patil et al. 2012).

**Fig. 9:**
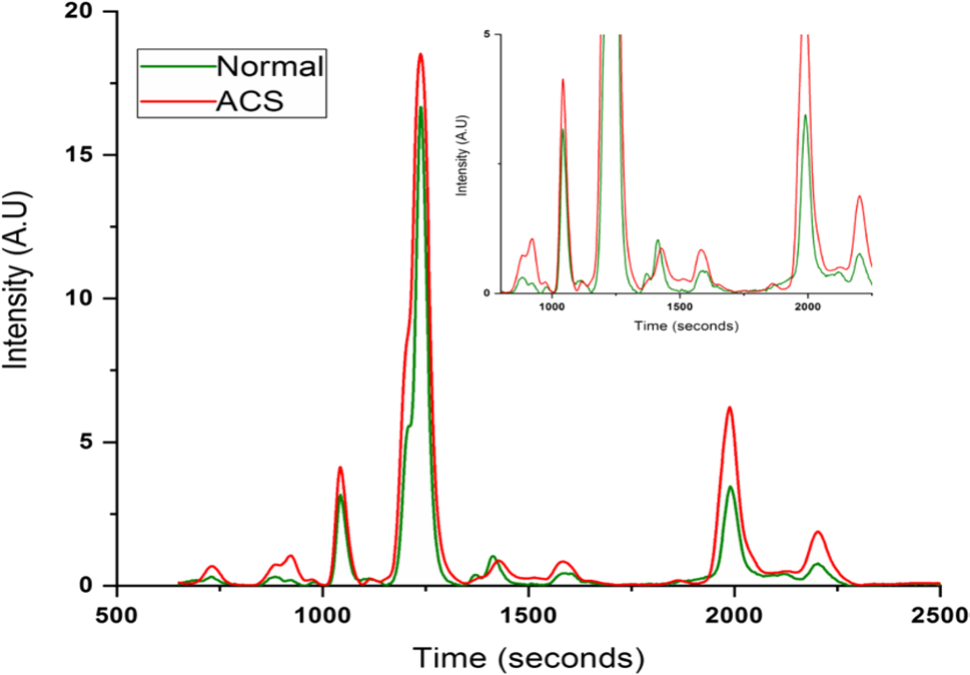
Averaged serum protein profile of normal and ACS. Inset: Protein profile in expanded scale

**Fig. 10:**
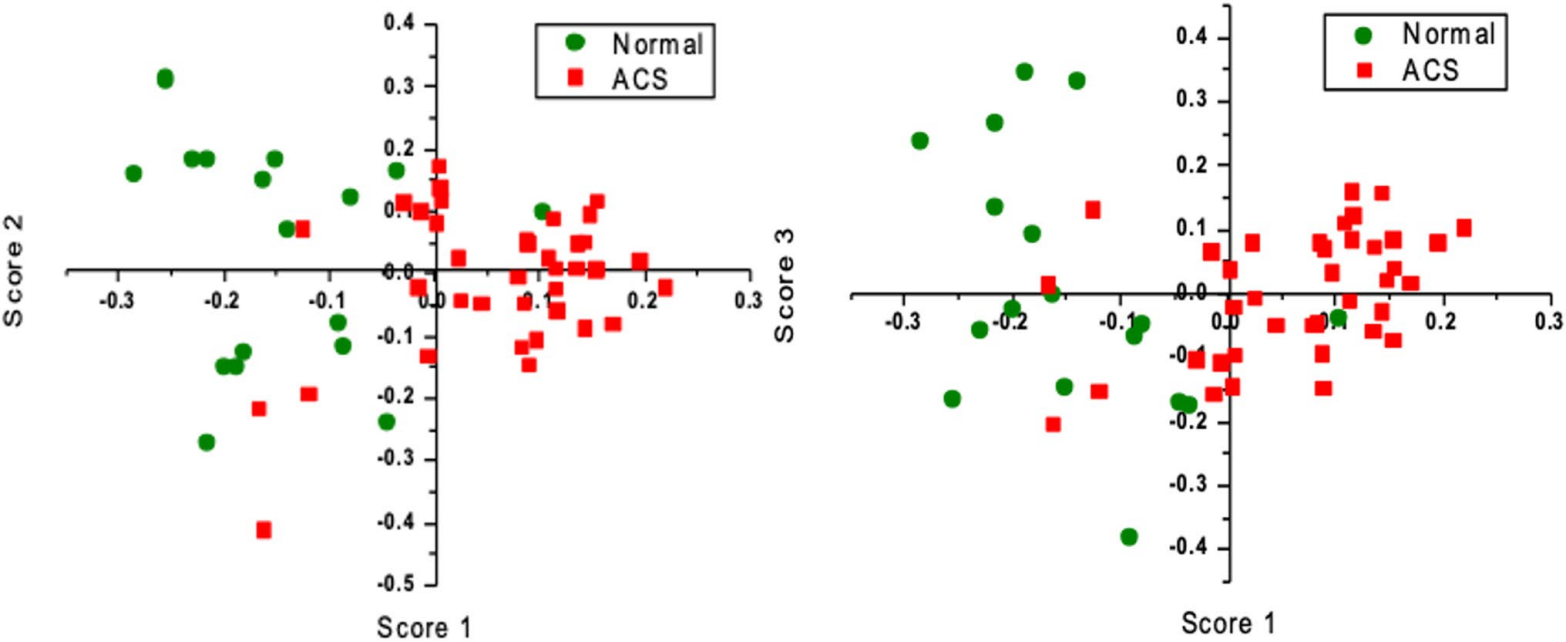
Principal component analysis results. **a** Sample scores of factor 1 versus scores of factor 2 and **b** score 1 versus score 3 (17 healthy and 32 ACS)

Averaged protein profiles of ACS and normal samples can be seen in Fig. 9 which suggests that there are significant variations in relative intensities. Preliminary data processing by principal component analysis (PCA) has given very good discrimination between protein profiles of Normal and ACS (Fig. 10). The serum protein profile analysis gives a clear picture of the pathophysiological condition. The HPLC-LIF detection method gives a simple method, which can even allow visual discrimination between normal and abnormal samples, for early diagnosis. In the present study, PCA has given clear discrimination between ACS and normal samples with high specificity and sensitivity. Further detailed study of ACS whole blood samples is being continued using different techniques such as HPLC-LED-induced fluorescence (IF), Raman spectroscopy, fluorescence, etc.

**Fig. 11:**
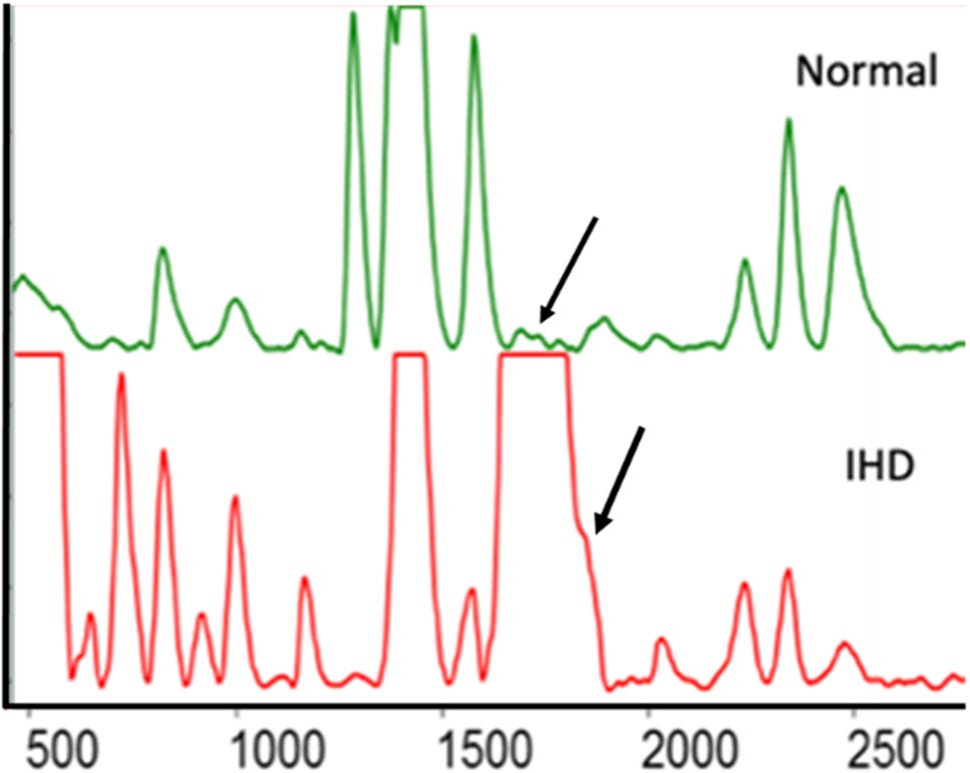
Region of serum chromatogram showing variation in the CPK peak (1800s) intensity

As a typical example of the application of Protein profiling in CVD early detection, the variation in CPK from a normal-smoker-IHD subject is shown in Fig. 11. CPK-MB is a biomarker for CVD, elevation of CPK in serum is found in case of inflammation as discussed before. Tobacco consumption is one of the risk factors for CVD. Studies have suggested CPK is observed only in extremely small amounts in normal serum, while in smokers it starts to show an increase (Sujatha et al. 2006). In IHD—ischemic heart disease, its concentration in serum exceeds even that of the most abundant serum protein, HSA. Monitoring of CPK can thus serve as an efficient method for early onset CVD conditions in even regular smokers, where the marker starts to show an increase from normal.

#### Discussion

The delay in getting medical attention to CVD patient is a major influence on patient condition and long-term health. This situation can be minimized with the help of early or fast diagnosis of the disease. Troponin is considered to be the gold standard biomarker for CVD. Because of its poor sensitivity with current methods, there is an urgent need for reliable biomarker/biomarkers, which should be specific to the type of CVD, sufficiently sensitive, and easy to detect by clinicians. In this regard, multi marker detection combined with pattern analysis is found to be effective. Detection of markers representing each pathophysiological (inflammation, stress, plaque instability, etc.) condition of the disease may be highly desirable. One of the approaches to achieve this goal is by developing a highly sensitive method for the detection of biomarkers in body fluids such as serum, saliva, etc. The advancement in chromatographic techniques, mass spectrometry, capillary electrophoresis, nano material-based detection method, etc. has opened the doors for the detection of multiple biomarkers. Most of these techniques are still laboratory based, have poor reproducibility outside lab condition measurements, are costly, need complicated experimental setup and hence professionals to operate, etc. There is thus an urgent need for user-friendly, low-cost, simple systems with techniques for simultaneous detection of multiple biomarkers with high sensitivity and specificity, to enable more precise data analysis using Artificial Intelligence and Machine Learning methods, so that operation and diagnosis can be effective, operator-independent, needing only trained technicians that can be adaptable in hospitals and clinics. HPLC combined with laser-induced fluorescence technique can be a potential tool to analyze serum protein profiles with multi-marker diagnosis of CVDs by AI/ML methods. It should also be noted here that in addition to screening, early detection, and therapy follow-up, the technique can be used to establish the identity of the marker proteins, either by running the selected tumor markers in a co-injection technique (Patil et al. 2012) or recording the Raman/SERS spectra of the collected fractions and comparing with Spectral Data Banks.
